# A correction to the bandwidth difference method for assessment of magnetic resonance static field homogeneity

**DOI:** 10.1002/mp.70673

**Published:** 2026-09-15

**Authors:** Joseph Wishart, Christina L. Brunnquell, Daniel Vergara, Jason Ostenson

**Affiliations:** ^1^ Department of Radiology University of Washington Seattle Washington USA; ^2^ Department of Radiology University of Minnesota Minneapolis Minnesota USA

**Keywords:** magnetic resonance imaging, quality control, static magnetic field homogeneity

## Abstract

**Background:**

Published guidance offers four methods of static field inhomogeneity $\left(\Delta B_{0}\right)$ measurement, including a bandwidth difference method. However, an error exists in the implementation of this method, which we demonstrate analytically and experimentally.

**Purpose:**

To propose and validate a correction to the bandwidth difference method for quantifying $\Delta B_{0}$.

**Methods:**

We outline a correction in the method for quantifying local $\Delta B_{0}$ by measuring spatial translations rather than diameter measurements between MRI images with different receive bandwidths. To validate the approach, we acquired images of a cylindrical phantom containing a fixed array of fluid‐filled pins. We adjusted static field shims to create approximately linear and quadratic field perturbations and acquired high‐ and low‐bandwidth spoiled gradient echo sequences with anterior‐posterior (AP) and left‐right (LR) frequency‐encoding. We then measured translations of centroid pin locations between low‐ and high‐bandwidth images. Finally, we propose and validate an implementation strategy using a spherical phantom. In both cases, a vendor‐provided field mapping sequence provided reference homogeneity measurements.

**Results:**

Under manually adjusted shim settings, root‐mean‐square (RMS) and peak‐to‐peak (PP) $\Delta B_{0}$ were 2.46 and 6.29 parts per million (PPM) respectively, measured with the field mapping technique. Results of the proposed bandwidth difference method showed strong agreement; RMS and PP $\Delta B_{0}$ were 2.38 and 6.22 PPM respectively for AP frequency‐encoding and 2.38 and 6.08 PPM respectively for LR frequency‐encoding. In a spherical phantom, the proposed method agreed with reference measurements to within 0.25 PPM (< 7%). However, PP $\Delta B_{0}$ was underestimated using the diameter‐based method—in a spherical phantom, measured $\Delta B_{0}$ was 0.74 PPM compared to 3.52 and 3.76 PPM using the reference and revised methods, respectively.

**Conclusions:**

This work experimentally validates a revised bandwidth difference approach for $\Delta B_{0}$ quantification, showing that it remains sensitive under spatially nonlinear $\Delta B_{0}$; the diameter‐based method is shown to be inaccurate for such cases.

## 1 INTRODUCTION

Signal localization and resulting image formation in MRI are principally determined through measurements of spatially dependent precessional frequencies, governed by the Larmor equation:

(1) $$\omega \left(x,y,z\right)=\gamma \cdot \left(B_{0}+\Delta B_{0}\left(x,y,z\right)\right)$$

where ω is the angular frequency of precession, $B_{0}$ is the reference static magnetic field strength, $\Delta B_{0}$ is the static field inhomogeneity relative to $B_{0}$, and γ is the gyromagnetic ratio ($\gamma /\left(2\pi \right)\approx 42.58\;\mathrm{MHz}\;T^{-1}$ for protons). To encode one dimension of spatial position, a linear magnetic field gradient G is applied along a frequency‐encoding direction, for example, x. This produces a continuum of frequencies that varies linearly with x according to the following expression:

(2) $$\omega \;\left(x,y,z\right)=\gamma \cdot \left(B_{0}+\Delta B_{0}\left(x,y,z\right)+G\cdot x\right)$$

Equation 2 implies that field inhomogeneity, a function varying in three dimensions, directly affects the local frequency and therefore measured position of objects in the frequency‐encoding direction. An inhomogeneous static field can lead to geometric distortion, signal degradation due to reduced T2*, poor fat suppression, inadequate frequency‐selective excitation, and broadened spectral peaks in spectroscopy.^1^, ^2^, ^3^, ^4^ Therefore, accurate quantification of $\Delta B_{0}$ is essential for identifying these issues before they affect patient care. In clinical MRI systems, $\Delta B_{0}$ is quantified by deviations relative to the system's determination of the Larmor frequency ($\omega_{0}$), which is typically defined as the mode of the frequency spectrum acquired during pre‐scan. So, $B_{0}=\omega_{0}\;/\gamma$ and $\Delta B_{0}=\Delta \omega_{0}\;/\gamma$ for frequency deviations $\Delta \omega_{0}$ from $\omega_{0}$. $\Delta B_{0}$ is specified within a diameter of spherical volume (DSV), typically 15–45 cm.

Scanners must be optimized to not only create a uniform static field but also modulate existing fields within the bore to re‐establish field homogeneity. Field homogeneity is perturbed by objects within and around the magnet based on the magnetic properties of the objects’ composition.^5^ These inhomogeneities are corrected with passive and active magnetic shims internal to the MRI system. Linear field corrections are applied as fixed active shims by the gradient system, whereas passive shims placed at installation and additional active coil shims provide higher order corrections.^6^, ^7^ Without correctly functioning shims, field inhomogeneities persist, potentially leading to one or more of the artifacts previously mentioned.

Annual evaluation of $\Delta B_{0}$ is required for accreditation from the American College of Radiology (ACR), The Joint Commission (TJC), RadSite, and Intersocietal Accreditation Commission. Although TJC and the ACR do not provide action thresholds,^8^ vendors often supply relevant performance criteria. Additionally, the ACR gives example cases when qualitative evaluation or longitudinal comparisons of test results identified local field contamination from external objects that might otherwise go unnoticed.^8^

$\Delta B_{0}$ is characterized as the root‐mean‐square (RMS) or peak‐to‐peak (PP) deviation from the Larmor frequency over a specified DSV and is typically given in Hz or parts per million (PPM). General tolerance recommendations are provided in American Association of Physicists in Medicine (AAPM) Report No. 100: for a 35‐cm DSV, acceptance criteria are < 0.5 PPM RMS for routine systems and < 0.1 PPM RMS for systems that perform echo‐planar imaging and/or spectroscopy.^9^

Techniques for $\Delta B_{0}$ quantification are included in the 2015 ACR MRI Quality Control (QC) Manual and were recently compiled in AAPM Task Group Report No. 325 (TG 325).^8^, ^10^ One of the four methods described is the bandwidth difference method, first presented by Chen et al. in 2006.^11^ $\Delta B_{0}$ quantification using the bandwidth difference method is based on geometric distortion between phantom images acquired at or near the minimum and maximum receiver bandwidths allowed by the scanner. However, procedures outlined in TG 325 and the ACR MRI QC Manual suggest making distance measurements across the diameter of the phantom, which differs from the point‐based position assessment originally presented by Chen et al. in 2006.^11^ As will be shown, this is an important difference, potentially leading to a mischaracterization of $\Delta B_{0}$.

This work seeks to correct the guidance for proper implementation of the equations that describe the bandwidth difference method. It comprises three components: a mathematical derivation of the original method and correction of its implementation, an experimental validation of the revised implementation, and a presentation of a potential implementation strategy in a standard spherical phantom.

## 2 METHODS

### 2.1 Derivation of bandwidth difference method

In this subsection, we derive an expression for the field inhomogeneity as a function of the displacement of fiducials between images acquired at two different receive bandwidths.

Assuming a uniform frequency‐encoding gradient G along a field‐of‐view (FOV), it follows that:

(3) $$G=\frac{2\pi }{\gamma }\;\cdot \frac{BW}{FOV}$$

where BW is the receive bandwidth. To connect Equation 3 to $\Delta B_{0}$, we note that an MRI acquisition using a frequency‐encoding gradient with amplitude $G_{j}$ will experience distortion in the frequency‐encoding direction such that an object at $x_{i}$ will appear at:

(4) $$x_{i,j}=x_{i}+\frac{\Delta B_{0}\left(x_{i}\right)}{G_{j}}$$

where $\Delta B_{0}\left(x_{i}\right)$ represents the *local* static field inhomogeneity at position $x_{i}$ relative to the reference resonance frequency.

By combining Equations 3 and 4 for acquisitions at receive bandwidths $BW_{1}$ and $BW_{2}$, we obtain an expression analogous to that proposed by Chen et al. in 2006 (see their Equation 4), which forms the basis of the bandwidth difference method.^11^

(5) $$\Delta B_{0}\left(x_{i}\right)=\frac{2\pi \cdot BW_{1}\cdot BW_{2}\cdot \left(x_{i,1}-x_{i,2}\right)}{\gamma \cdot FOV\cdot (BW_{2}-BW_{1})}\;$$

Note, subscripts 1 and 2 indicate different acquisitional bandwidths whereas subscript *i*, here and in subsequent equations, indicates fiducial position. Importantly, Equation 5 defines local inhomogeneity as a function of the displacement of $x_{i}$ between acquisitions at different bandwidths. However, established guidance^8^, ^10^ recommends an approach using phantom diameter measurements rather than positional markers, as described below.

**FIGURE 1:**
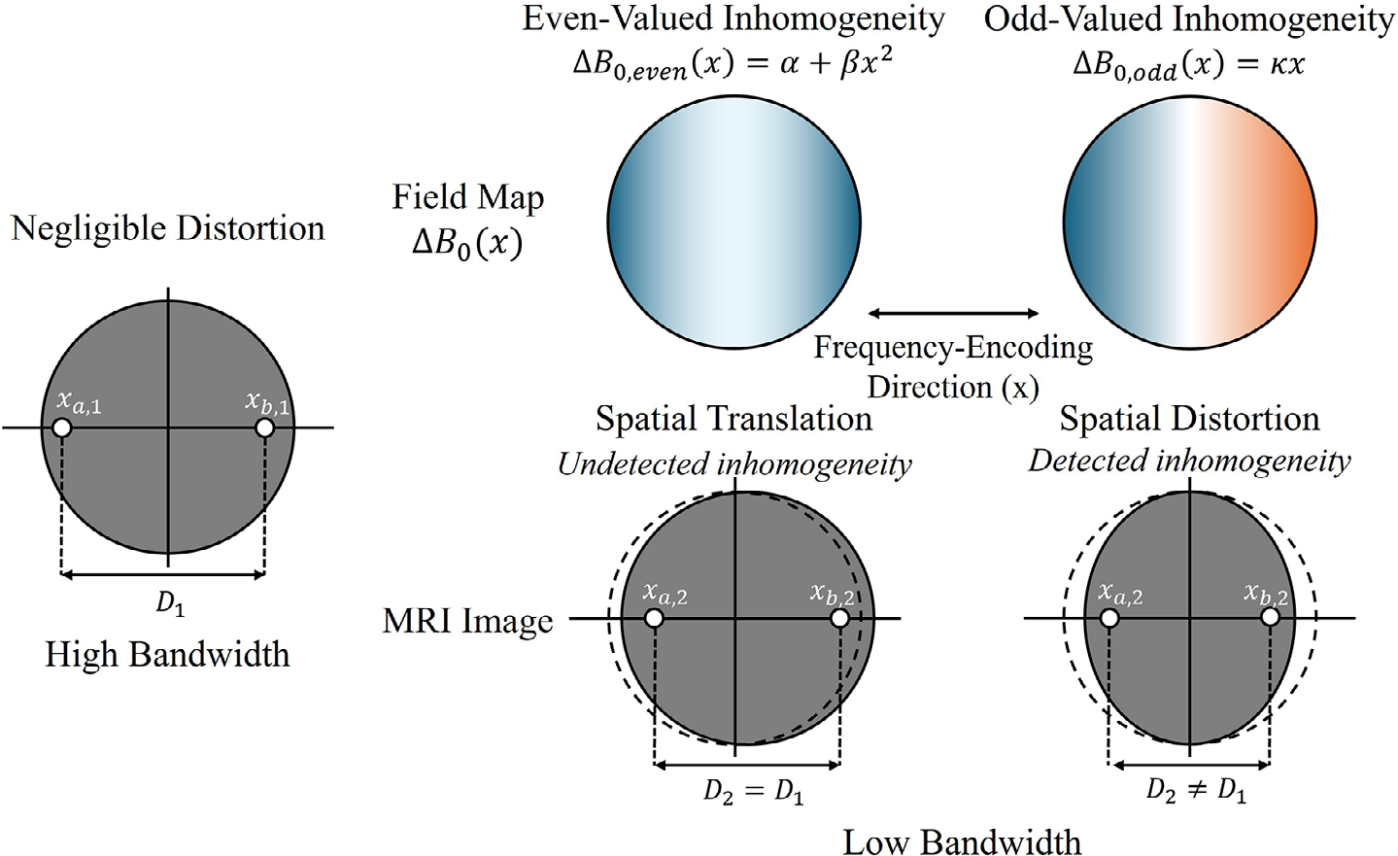
Diagram visualizing shifts in position of fiducials with two functional forms (quadratic/even and linear/odd) of field distortion. Colored circles display even and odd $B_{0}$ inhomogeneity profiles ($\Delta B_{0}$). Blue and orange colors indicate field strengths below and above nominal while white indicates zero inhomogeneity. Gray circles illustrate reconstructed phantom images, and dashed lines outline the true shape of the phantom. An image acquired with high bandwidth shows negligible distortion (left), whereas low bandwidth images yield phantom translation and distortion under even and odd inhomogeneity profiles respectively (lower left, right). Notably, Equation 9 incorrectly suggests zero inhomogeneity for an even functional form of field inhomogeneity due to equal distance measurements ($D_{1}=D_{2}$) between low‐ and high‐bandwidth acquisitions.

Consider a uniform phantom with two fiducial markers located at physical coordinate positions $x_{a}$ and $x_{b}$. Acquisitions with receive bandwidths $BW_{1}$ and $BW_{2}$ will result in visualization of the fiducials at locations $x_{a,1}$, $x_{b,1}$ and $x_{a,2}$, $x_{b,2}$ respectively (see Figure 1). Suppose images 1 and 2 are acquired with low and high bandwidth, respectively. Using Equation 4, the distances between fiducials a and b measured in images with bandwidths $BW_{1}$ and $BW_{2}$ are:

(6) $$D_{1}=x_{a,1}\;-\;x_{b,1}=x_{a}\;-x_{b}+\frac{\Delta B_{0}\left(x_{a}\right)-\Delta B_{0}\left(x_{b}\right)}{G_{1}}$$

(7) $$D_{2}=x_{a,2}\;-\;x_{b,2}=x_{a}\;-x_{b}+\frac{\Delta B_{0}\left(x_{a}\right)-\Delta B_{0}\left(x_{b}\right)}{G_{2}}$$

Subtracting Equation 7 from Equation 6 and rearranging using Equation 3 yields:

(8) $$\Delta B_{0}\left(x_{a}\right)-\Delta B_{0}\left(x_{b}\right)=\frac{2\pi }{\gamma \cdot FOV}\;\cdot \frac{D_{1}-D_{2}}{\frac{1}{BW_{1}}\;-\;\frac{1}{BW_{2}}}$$

Importantly, Equation 8 does not explicitly quantify $\Delta B_{0}$ but rather the difference in local $\Delta B_{0}$ values at points $x_{a}$ and $x_{b}$.

To illustrate the shortcoming of Equation 8, consider an inhomogeneity profile that follows an even‐valued function (Figure 1, center). Such an example is a quadratic function of the form: $\Delta B_{0,even}\;\left(x\right)=\alpha +\beta x^{2}$. By convention, isocenter is located at x=0 where the inhomogeneity is minimized. Taking $x_{a}$ and $x_{b}$ to be points on opposite edges of a phantom (i.e., $x_{a}=-x_{b}$), it follows that $\Delta B_{0,even}\;\left(x_{a}\right)=\Delta B_{0,even}\left(x_{b}\right)$. Evaluating Equation 8 then implies that $D_{1}=D_{2}$, even if $\Delta B_{0,even}\;\left(x_{a}\right)=\Delta B_{0,even}\left(x_{b}\right)\neq 0$.

Conversely, consider $\Delta B_{0}$ having an odd‐valued, linear function of x (Figure 1, right), $\Delta B_{0,odd}\;\left(x\right)=\kappa x$ where $\Delta B_{0,odd}\;\left(x_{a}\right)=-\Delta B_{0,odd}\left(x_{b}\right)$. Because inhomogeneity typically worsens with increasing distance from isocenter,^10^ PP $\Delta B_{0}$ for $\Delta B_{0,odd}\left(x\right)$ can be represented by the difference between local inhomogeneities at phantom edges: PP $\Delta B_{0,\mathrm{odd}}=\Delta B_{0,\mathrm{odd}}\left(x_{a}\right)-\Delta B_{0,\mathrm{odd}}\;\left(x_{b}\right)=2\Delta B_{0,\mathrm{odd}}\left(x_{a}\right)$. Applying this specific case to Equation 8, after simplifying and converting to units of PPM, one arrives at the expression presented in guidance documents (Equation 9 in TG 325, p. 74 in ACR MRI QC Manual):

(9) $$\frac{\Delta B_{0}}{B_{0}}\;\left(PPM\right)=\frac{BW_{1}\cdot BW_{2}\cdot \left(D_{1}-D_{2}\right)}{f_{center}\cdot FOV\cdot \left(BW_{2}-\;BW_{1}\right)}\;$$

The arguments presented yield two important conclusions. First, equivalent distance measurements for successive acquisitions with low and high bandwidths can erroneously imply zero inhomogeneity when $\Delta B_{0}\;\left(x_{a}\right)=\Delta B_{0}\;\left(x_{b}\right)\neq 0$. Second, Equation 9 does not quantify RMS $\Delta B_{0}$ (as suggested in TG325) but instead represents the difference in local $\Delta B_{0}$ between the end points of the distance measurements.

Assuming a conventional definition where PP $\Delta B_{0}=\Delta B_{0,\mathrm{Max}}-\Delta B_{0,\mathrm{Min}}$, Equation 9 is equivalent to PP $\Delta B_{0}$ if and only if the $\Delta B_{0}$ extrema are located at these end points. As shown in the above illustration, this condition is satisfied by a function that is odd along the frequency‐encoding axis. In fact, the function need not be entirely odd; Equation 9 is valid for any case in which the extrema of $\Delta B_{0}$ are captured by the points evaluated. However, even under these conditions, the result is only relative to the dimension evaluated, not throughout the entire DSV. Additionally, such assumptions are not universally applicable without prior knowledge of the functional form of $\Delta B_{0}$. For example, if the locations of $\Delta B_{0}$ extrema are located on diagonal positions of the phantom and distance measurements are performed only along the AP and LR directions, extrema will not be captured in the measurement and quantification will be biased.

Valid universal implementation of the bandwidth difference method requires measuring positional shifts of many fiducial markers throughout a DSV, applying Equation 5 to quantify local $\Delta B_{0}$, then calculating PP and RMS metrics. Such a procedure is presented below as experimental validation.

### 2.2 Experimental validation

To validate Equation 5, a series of image acquisitions was performed on a 3.0 T MRI scanner (MR 7700; Philips, Best, The Netherlands). Images of a 40 cm diameter cylindrical phantom were acquired. This vendor‐provided phantom contains a fixed array of fluid‐filled pins. To accentuate the effects of field perturbations on reconstructed phantom geometry, static field shim settings were adjusted to create approximately linear and quadratic modulations in $B_{0}$ in the AP and LR directions respectively. A $\Delta B_{0}$ field map was reconstructed from a vendor‐provided sequence, yielding an inhomogeneity image measured in Hz relative to the center frequency. This image was scaled to represent $\Delta B_{0}$ in PPM by dividing by the center frequency and used as the reference $\Delta B_{0}$ measurement. A corresponding magnitude image was used to identify the spatial locations and geometric properties of individual pins using custom image analysis implemented in MATLAB R2025b (MathWorks; Natick, MA). Magnitude images were intensity‐scaled and thresholded to separate fluid‐filled pins from background. Pins were then identified using a connected‐component algorithm with 8‐connected neighborhoods, generating centroid locations and circularity measures for all pins. Using the identified pin regions from the magnitude image as a mask, mean $\Delta B_{0}$ values were calculated within each identified pin region of the field map.

Low and high bandwidth spoiled gradient echo (SPGR) sequences were acquired with frequency‐encoding in AP and LR directions. Imaging parameters are provided in Table 1. The centroid pixel locations of phantom pins in high and low bandwidth images were obtained using the same MATLAB algorithm described above. Displacements of centroid pin locations between high and low bandwidth acquisitions were calculated in pixels and converted to mm using known pixel spacing. Local $\Delta B_{0}$ values were then calculated at each pin location using Equation 5.

**TABLE 1:** Acquisition parameters for scanning cylindrical (Experimental Validation) and spherical (Revised Approach) phantoms for bandwidth difference and field map methods.

	Experimental Validation		Revised Approach	
Image	Field Map^*^	BW (Low, High)	Field Map^*^	BW (Low, High)
Sequence	Dual‐Echo SPGR	SPGR	Dual‐Echo SPGR	SPGR
Bandwidth(s) (Hz/Pixel)	473	83, 473	992	46, 703
Matrix (AP x LR)	800 × 800	800 × 800	352 × 352	480 × 480
Field of View (mm)	400	400	350	350
Slice Thickness (mm)	5	5	5	5
TE(s) (ms)	7.5, 8.5	7.5	2.2, 3.2	12
TR (ms)	30	30	10	25
Averages	4	2, 4	2	2, 8
Flip Angle (deg)	60	60	10	10

Abbreviations: BW, Bandwidth Difference; SPGR, spoiled gradient echo.

* The vendor field map is a phase subtraction technique.

Values of $\Delta B_{0}$ in PPM were calculated at each pin location for the field map and revised bandwidth difference methods. To assess agreement between the reference field map method and revised bandwidth difference method, Bland‐Altman analysis was performed; for each pin, the difference in measured $\Delta B_{0}$ between the two methods was plotted as a function of the mean $\Delta B_{0}$ of the two methods.

To demonstrate the shortcoming of the diameter‐based bandwidth difference method, values of $\Delta B_{0}$ were calculated using Equation 9 where $D_{j}$ represents the phantom diameter measured in magnitude images acquired with bandwidth *j*. Measurements were performed in AP and LR directions under corresponding directions of frequency‐encoding.

### 2.3 Revised approach for a uniform phantom

The technique described above is limited in its practicality, necessitating a phantom not available on all systems and custom code or scripting. However, because field homogeneity typically worsens with increasing distance from isocenter,^10^ an approximation of inhomogeneity can be made using phantom boundaries in subtraction images. This technique yields local inhomogeneity at phantom edges and therefore cannot be used to compute RMS $\Delta B_{0}$ or its functional form. However, the method is simple and yields accurate $\Delta B_{0}$ measurements at locations likely to indicate maximum inhomogeneity for most profiles encountered in clinical magnets. The approach is presented below as a potential implementation strategy, validated in a spherical phantom. The selection of a spherical phantom is intentional since a spherical phantom will perturb the static field inside the sphere much less than a short cylindrical phantom sitting coaxially with the static field direction.

**FIGURE 2:**
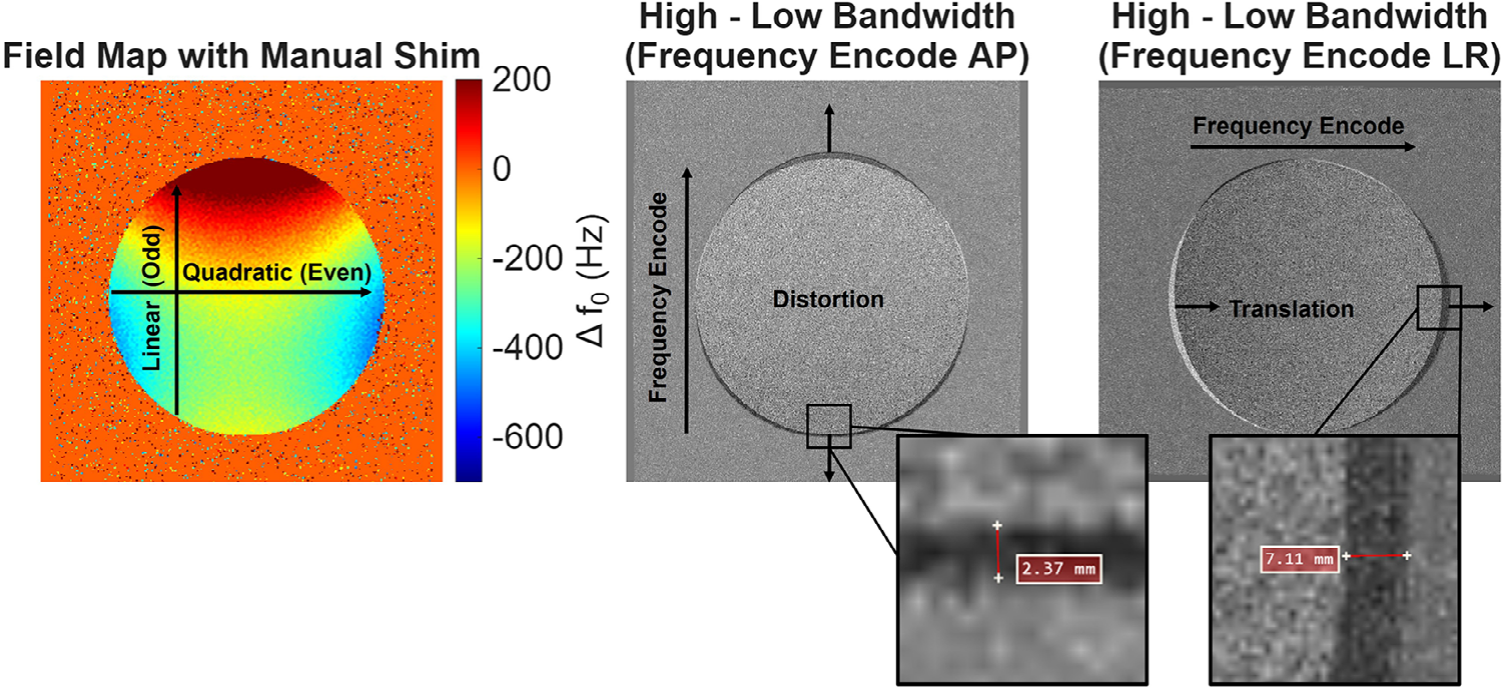
Demonstration of practical implementation in a spherical phantom. Adjusting static field shims to induce exaggerated inhomogeneity, a field map was produced (left) visualizing approximately even and odd $\Delta B_{0}$ in LR and AP directions respectively. Distortion (center) and translation (right) are induced at low bandwidth for odd and even $\Delta B_{0}$ respectively. By measuring phantom edge shifts in subtraction images (boxes), local $\Delta B_{0}$ can be calculated using Equation 5.

Using parameters shown in Table 1 and shim perturbations similar to those used in the validation, field maps and bandwidth difference images were acquired through the central axial plane of a uniform 24 cm diameter spherical phantom filled with MARCOL 82 oil mixed with MACROLEX blue dye (0.011 g per 1000 mL). From a subtraction image (vendor‐specific instructions for image algebra are available in TG 325 resources^12^), maximum displacements of phantom edges were measured (Figure 2). Equation 5 was then applied to estimate local $\Delta B_{0}$, where $x_{i,1}-x_{i,2}$ represents the measured edge displacement. The relative signs of $\Delta B_{0}$, indicated by shift direction and resulting grayscale in subtraction images, are important for PP estimation. For proper comparison, signs of $\Delta B_{0}$ using the revised method were matched to the convention used in the field map.

To validate this approach, $\Delta B_{0}$ measurements were compared to mean values within small (∼50 $mm^{2}$) regions of the field map near phantom edges. To compare with the incorrect diameter‐based BW difference method, PP $\Delta B_{0}$ was computed from phantom diameter measurements using Equation 9. Directional PP $\Delta B_{0}$ was calculated as the difference between estimated values of $\Delta B_{0,Max}$ and $\Delta B_{0,Min}$ in each frequency‐encoding direction (e.g., AP) and nominal PP $\Delta B_{0}$ was calculated in the same manner using maximum and minimum values from the entire image. Limitations to these calculations are further discussed in Results and Discussion sections.

## 3 RESULTS

**FIGURE 3:**
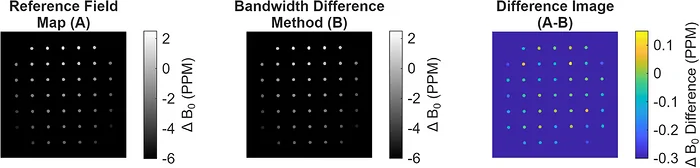
$\Delta B_{0}$ maps for phantom pin locations obtained via a vendor field mapping sequence (A) and proposed bandwidth difference method (AP frequency‐encoding) using pin centroid locations (B) and a subtraction image of the two methods to visualize agreement.

Results of $\Delta B_{0}$ quantification through pixel centroid measurement and comparison to field mapping are shown in Figure 3. For the field mapping quantification method, displayed pin values are the mean inhomogeneity values within each pin location obtained from the field map. For the bandwidth difference method, displayed pin values were calculated using Equation 5 where $x_{i,j}$ represents centroid location *i* from acquisition bandwidth *j*, measured using MATLAB‐based image processing. Displayed values are from images acquired under AP frequency‐encoding, although a similar map was obtained using LR encoding.

**FIGURE 4:**
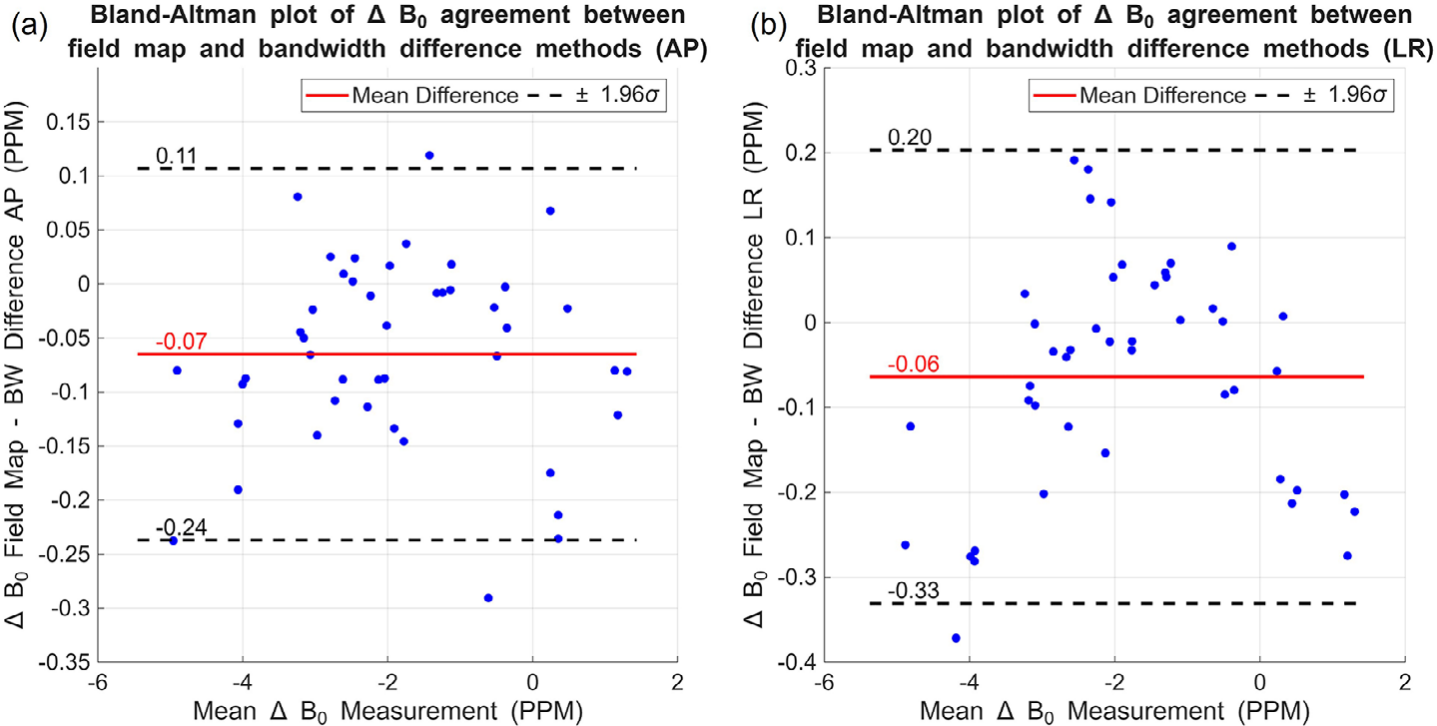
Bland‐Altman plots for $\Delta B_{0}$ quantification show relative agreement between the field mapping sequence and proposed bandwidth difference methods for anterior‐posterior (AP) (a) and left‐right (LR) frequency‐encoding (b). Red lines represent mean differences between $\Delta B_{0}$ quantification methods, while dashed lines represent the limits of agreement.

For each pin location, the difference between field mapping and bandwidth difference quantification was calculated and plotted against the mean $\Delta B_{0}$ between methods. Bland‐Altman plots are shown for AP and LR frequency‐encoding in Figure 4.

Under exaggerated inhomogeneous conditions intentionally generated from suboptimal shimming, the measured RMS and PP $\Delta B_{0}$ among pin measurements using the reference field mapping sequence were 2.46 and 6.29 PPM respectively. Using the proposed bandwidth difference method, RMS and PP $\Delta B_{0}$ were measured to be 2.38 and 6.22 PPM respectively for AP frequency‐encoding and 2.38 and 6.08 PPM respectively for LR frequency‐encoding. Using Equation 9 and measured diameters in the cylindrical phantom, PP $\Delta B_{0}$ was 5.09 and 0.13 PPM for AP and LR frequency‐encoding, respectively.

Results from the revised approach for a uniform spherical phantom are shown in Table 2. The mean absolute difference in local $\Delta B_{0}$ values between the revised approach and field map was 0.14 PPM (5.01%).

**TABLE 2:** Comparison of local and PP $\Delta B_{0}$ measurements obtained from the field map, revised bandwidth difference, and diameter‐based measurement methods in a uniform spherical phantom. Nominal PP values were computed for global estimation under assumptions presented in the discussion.

Location	Field Map (PPM)	Revised BW Method (PPM)	Diameter‐Based Method (PPM)^†^
Anterior	2.98	3.09	n/a
Posterior	−1.33	−1.25	n/a
Left	−3.26	−3.40	n/a
Right	−3.52	−3.76	n/a
Anterior‐Posterior PP	4.31	4.34	4.42
Left‐Right PP	3.52	3.76	0.74
Nominal PP	6.50	6.85	4.42^*^

^†^ Local $\Delta B_{0}$ cannot be determined using the diameter‐based method and are therefore listed as “n/a”.

* The diameter‐based method does not provide relative signs of $\Delta B_{0}$; the global PP estimate is assumed to take the maximum value among the dimensions evaluated.

## 4 DISCUSSION

The bandwidth difference method for $\Delta B_{0}$ quantification was originally established using point‐based measurements, though initial analysis was limited to PP quantification for a small DSV (22.6 cm).^11^ This methodology was incorrectly implemented in subsequent testing guides which suggest measuring changes in a phantom's diameter. The persistence of this error could be due to the commonality of odd‐valued inhomogeneity profiles for which the guidance is approximately valid, or simply due to the lack of clinical validation of different quantification techniques. This work expands on the source publication by evaluating $\Delta B_{0}$ throughout a large FOV and rectifies an error in established guidance.

Bland‐Altman plots show strong agreement between the proposed bandwidth difference and field map methods. As indicated in Figure 4, the proposed method overestimated $\Delta B_{0}$ by 0.07 and 0.06 PPM on average relative to the field map technique for AP and LR frequency‐encoding respectively. Qualitatively, there appears to be no strong functional dependence of method agreement on the magnitude of $\Delta B_{0}$ quantification. Additionally, the intervals between mean differences and limits of agreement (± 0.18 and ±0.26 PPM) represent just 2.9% and 4.2% of mean PP $\Delta B_{0}$, contain zero difference, and are well below reported standard deviations and biases among various methods documented in archived homogeneity data provided by the AAPM.^13^ RMS and PP quantification showed similarly strong agreement, yielding maximum differences of 0.08 and 0.21 PPM (3.25% and 3.34%) respectively.

As expected, using Equation 9 and cylindrical phantom diameter measurements significantly underestimated PP $\Delta B_{0}$ for the approximately quadratic inhomogeneity profile (LR frequency‐encoding), yielding 0.13 PPM compared to 6.29 PPM and 6.08 PPM using the field map and proposed pin‐based method respectively. However, the diameter measurements sample just two points on the phantom periphery, whereas the pin‐based measurements sample $\Delta B_{0}$ throughout the phantom. As such, values cannot be directly compared between methods.

Using the revised method in a uniform phantom, the mean absolute difference in local $\Delta B_{0}$ between the proposed implementation and reference field map was 0.14 PPM or 5.01%. The BW difference method overestimated local $|\Delta B_{0}|$ at three out of the four locations, likely reflecting differences in sampling location. Field map ROIs were positioned just inside the boundary, whereas the BW difference method samples the boundary itself, where $|\Delta B_{0}|$ is expected to be slightly larger. However, this difference is comparable to measurement uncertainty: standard deviations within field map ROIs were on average 15.6 Hz or 0.12 PPM. Edge displacement measurements may cause increased variability in $\Delta B_{0}$. Evaluating Equation 5 with $x_{i,1}-x_{i,2}$ equal to the pixel width (0.73 mm) yields an uncertainty 0.39 PPM. Overall, the observed differences between methods are within measurement uncertainties.

Importantly, the revised method provides local evaluations of $\Delta B_{0}$ whereas PP $\Delta B_{0}$ is defined as the difference between global maximum and minimum values of $\Delta B_{0}$. Consequently, the revised method provides an accurate measurement of PP $\Delta B_{0}$ only if sampled locations include the global extrema of $\Delta B_{0}$. For example, if measurements contain both positive and negative $\Delta B_{0}$ values, PP $\Delta B_{0}$ can be calculated as the difference between the maximum and minimum measurements, assuming $\Delta B_{0}$ does not go outside these extrema within the phantom. However, if $\Delta B_{0}$ extrema are located elsewhere, that is, on diagonal edges of the phantom, standard AP and LR measurements will fail to capture such inhomogeneity, leading to underestimation in PP $\Delta B_{0}$. Alternatively, if measurements share the same sign, PP $\Delta B_{0}$ is calculated as the maximum absolute value of measurements obtained at phantom edges. This assumes the minimum absolute value of $\Delta B_{0}$ (${|\Delta B_{0}|}_{min})$ is zero, an assumption that may be untrue. For example, cylindrical phantoms with their central axis parallel to the static field exhibit radially increasing $|\Delta B_{0}|$ with nonzero ${|\Delta B_{0}|}_{min}$ even when the static field is perfectly homogenous. In such cases, PP $\Delta B_{0}$ quantification will be biased depending on the sign of ${|\Delta B_{0}|}_{min}$. Although this supports the use of spherical phantoms, the revised method provides a practical means of identifying gross field perturbations at the boundaries of any shaped phantom.

To directly compare the revised approach relative to the diameter‐based method in the spherical phantom, we first evaluate PP homogeneity in AP and LR directions, separately. Equation 9 yields PP $\Delta B_{0}$ of 4.42 and 0.74 PPM for AP and LR frequency‐encoding, respectively. The AP‐direction PP estimate agrees well with the AP PP estimates using the field map and revised approach (4.31 and 4.34 PPM, respectively). This reflects the validity of the diameter‐based method for odd‐like geometry. Under the assumption that ${|\Delta B_{0}|}_{min}$ = 0, the PP estimates from LR frequency‐encoding (approximately even geometry) are 3.52 and 3.76 PPM using the field map and revised approach, respectively. Due to its insensitivity to translation, the diameter‐based method yields a biased estimate of 0.74 PPM. Globally, assuming the four measurements in Table 2 adequately capture the extrema of $\Delta B_{0}$, PP $\Delta B_{0}$ estimates would be 6.50 and 6.85 PPM using the field map and revised approach respectively. Conversely, the diameter‐based approach is insensitive to relative sign; a nominal PP estimate is taken to be the max PP $\Delta B_{0}$ among the dimensions evaluated, 4.42 PPM in this case. These results underscore the shortcoming of the diameter‐based method which significantly underestimates PP $\Delta B_{0}$.

Measurement uncertainty using the proposed method arises from the subjective assessment of phantom edges or distortion in phantom pin centroids, but such uncertainty can be mitigated with high‐resolution imaging and by performing measurements under high magnification, enabling accurate detection of the position of the edge of the phantom to within sub‐pixel accuracy. In our validation, the uncertainty (∼0.4 PPM) is smaller than clinically measured $\Delta B_{0}$ values from other homogeneity measurement methods archived by the AAPM: these range from 0.62 PPM (spectral peak, RMS, DSV < 30 cm) to 1.53 PPM (phase difference map, PP, variable DSV).^13^ This uncertainty is also smaller than the potential bias from the diameter‐based BW difference method.

In our multi‐pin validation, enhanced precision was achieved using centroid localization with MATLAB‐based image analysis. However, centroid measurements may be biased by geometric distortions of fiducial pins used for coordinate identification. Such distortions were readily visualized in low‐bandwidth images and are attributable to shim‐induced field inhomogeneity. Regardless, our validated method demonstrated accuracy and precision exceeding those achievable through phantom diameter measurements.

Another limitation of this study is the potential for confounding geometric disturbances caused by gradient nonlinearities and local field nonuniformities near phantom boundaries. These effects may be further modulated by differences in gradient strength imposed by varying bandwidths between acquisitions. However, modern scanners typically employ retrospective gradient linearity corrections based on spherical harmonic expansions up to fifth‐order terms, which are likely sufficient to mitigate inter‐acquisition variability.^14^ For this reason and to maintain consistency, we performed all measurements with gradient linearity corrections turned on. Additionally, consistent with literature recommendations, homogeneity assessments were performed only after evaluation of system gradient linearity.^8^, ^10^ Regarding field nonuniformities, quantification was performed in a sealed phantom, minimizing susceptibility interfaces and associated field disturbances.

Cylinders are suitable phantoms for homogeneity assessment, but can introduce additional distortions, as noted in TG 325. In this work, a cylindrical phantom was chosen to validate the proposed correction with relative measurements because of the fiducial markers found therein, rather than to serve as a recommended phantom. Cylindrical phantoms without internal fiducial markers can still be used with the revised method, but repeated measurements in three anatomical planes, with phantom repositioning, would be required.

Collectively, these limitations and potential confounders highlight the importance of performing $\Delta B_{0}$ quantification with a consistent methodology across longitudinal evaluations.

## 5 CONCLUSIONS

Current guidance for $\Delta B_{0}$ quantification using distance measurements is not accurate under all inhomogeneity geometries. This work provides a correction that remains sensitive to all inhomogeneity profiles. This is an important step; without consistent access to field mapping sequences for all MRI vendors, the bandwidth difference method remains one of the most universally deployable image‐based approaches to field homogeneity evaluation.

## CONFLICT OF INTEREST STATEMENT

The author declares no conflicts of interest.

## FUNDING INFORMATION

The authors have no funding to report.
